# Influence of Heart Rate Variability on Abstinence-Related Changes in Brain State in Everyday Drinkers

**DOI:** 10.3390/brainsci11060817

**Published:** 2021-06-20

**Authors:** Hope Peterson, Rhiannon E. Mayhugh, Mohsen Bahrami, Walter Jack Rejeski, Sean L. Simpson, Keri Heilman, Stephen W. Porges, Paul J. Laurienti

**Affiliations:** 1 Laboratory for Complex Brain Networks, Wake Forest University Health Sciences, Winston-Salem, NC 27104, USA; mbahrami@wakehealth.edu (M.B.); rejeski@wfu.edu (W.J.R.); slsimpso@wakehealth.edu (S.L.S.); plaurien@wakehealth.edu (P.J.L.); 2 Graduate Program, Wake Forest School of Medicine, Neuroscience, Winston-Salem, NC 27101, USA; 3 Professional Development and Career Office, Johns Hopkins University School of Medicine, Baltimore, MD 21287, USA; rhiannon.mayhugh@gmail.com; 4 Health and Exercise Science, Wake Forest University, Winston-Salem, NC 27109, USA; 5 Wake Forest School of Medicine, Biostatistics and Data Science, Winston-Salem, NC 27101, USA; 6 Department of Psychiatry, University of North Carolina School of Medicine, Chapel Hill, NC 27514, USA; heilmank@sbcglobal.net (K.H.); sporges@gmail.com (S.W.P.); 7 Department of Psychiatry, University of Illinois, Chicago, IL 60612, USA; 8 Wake Forest School of Medicine, Radiology, Winston-Salem, NC 27101, USA

**Keywords:** alcohol, brain networks, heart rate variability, abstinence, respiratory sinus arrhythmia

## Abstract

Alcohol consumption is now common practice worldwide, and functional brain networks are beginning to reveal the complex interactions observed with alcohol consumption and abstinence. The autonomic nervous system (ANS) has a well-documented relationship with alcohol use, and a growing body of research is finding links between the ANS and functional brain networks. This study recruited everyday drinkers in an effort to uncover the relationship between alcohol abstinence, ANS function, and whole brain functional brain networks. Participants (*n* = 29), 24–60 years-of-age, consumed moderate levels of alcohol regularly (males 2.4 (±0.26) drinks/day, females 2.3 (±0.96) drinks/day). ANS function, specifically cardiac vagal tone, was assessed using the Porges-Bohrer method for calculating respiratory sinus arrhythmia (PB_RSA_). Functional brain networks were generated from resting-state MRI scans obtained following 3-day periods of typical consumption and abstinence. A multi-task mixed-effects regression model determined the influences of HRV and drinking state on functional network connectivity. Results showed differences in the relationship between the strength of network connections and clustering coefficients across drinking states, moderated by PB_RSA_. Increases in connection strength between highly clustered nodes during abstinence as PB_RSA_ increases demonstrates a greater possible range of topological configurations at high PB_RSA_ values. This novel finding begins to shed light on the complex interactions between typical alcohol abstinence and physiological responses of the central and autonomic nervous system.

## 1. Introduction

Alcohol use is a leading risk factor for declining health and global disease burden [1], but cessation of alcohol consumption is highly associated with stress and craving which drive the risk of relapse [2,3,4,5]. Stress and craving responses include visceral sensations and central neural processes such as the drive to consume alcohol, a motive directly related to the reward or “wanting” properties of alcohol [6]. As described in Polyvagal Theory (PVT; [7,8]), the vagus nerve is the major bidirectional pathway of the nervous system linking the body to the brain. Through evolution, the ventral root of the vagus in mammals has evolved as a key biological structure supporting sociality in humans and providing a dynamic system of monitoring threat to the organism through a process termed nociception. Increased threat reduces cardiac vagal tone (CVT) which subsequently leads to a coping response orchestrated by the central nervous system. As described in PVT, coping often involves an “active” response which can involve cognition, emotion and/or behavior. For example, people consume alcohol as a means to reduce unpleasant states induced by stress. In extreme stress, such as trauma, research has shown that the vagal system can shift control to the dorsal root of the vagus nerve and induce a powerful “passive” form of coping known as the freeze response. Porges and Bohrer [9] have developed a valid metric of CVT, which represents activity in the ventral root of the vagus nerve and is abbreviated as PB_RSA._ This abbreviation is used to distinguish it from the peak-to-trough method for determining RSA, PT_RSA,_ which is common in the literature but is less desirable. Research has consistently shown that higher levels of CVT are associated with psychological resilience, improved physiological recovery following exposure to stressors [10], greater emotional flexibility [11], and most relevant to the current study, improved ability to cope with alcohol cravings [12]

Alcohol craving in patients with an alcohol use disorder (AUD) has been associated with high frequency heart rate variability (HF-HRV), another metric used to assess CVT, with acute consumption being related to decreasing HF-HRV [13,14,15,16]. Conceptually RSA is equivalent to HF-HRV when the frequency band is inclusive of the frequencies of spontaneous breathing and RSA is an accurate estimate of CVT when RSA is calculated with the PB_RSA_ metric. Alcohol craving ratings in the everyday drinking population are higher with lower dynamic PB_RSA_ [17]. Long-term alcohol use has also been associated with decreased HF-HRV with studies finding lower HF-HRV in alcohol dependent inpatients compared to age and gender matched controls [18,19,20]. However, only a moderate period of abstinence (i.e., 4 months) is needed to improve HF-HRV in individuals with AUD [20]. Another recent study has documented decreased PT_RSA_ in conjunction with greater anxiety and stress and overall poor mood in abstinence alcoholic men [21].

The relationship between CVT and everyday drinking has been less thoroughly explored, a shortcoming that is the focus of the current study. Specifically, our goal was to examine fundamental neural mechanisms underlying the influence of the autonomic nervous system (ANS) on brain responses to alcohol abstinence in regular drinkers. To do so, ANS activity was assessed using PB_RSA_ in an effort to capture the functional relationship between heart rate, respiration, and brainstem function [22,23]. We are aware that Thayer and colleagues [24,25] have proposed a hierarchical neurovisceral integrative (NVI) model to increase understanding of vagal control with both cognitive performance and emotional/physical health. However, in the current paper, we are examining whole brain functionality as opposed to regional specificity inherent to the NVI model. Our approach is consistent with the foundational research conducted by Nobel Laureate Walter Hess (1949) [26], who focused on a model of an integrated nervous system regulating both brain and body. Also, as described above, we have elected to use PB_RSA_ as a metric of CVT, since our work is based on PVT. PVT provides an adaptive hierarchical model of autonomic function that parallels phylogeny and maturation. It is this hierarchical model of autonomic regulation that is disrupted by AUD.

The majority of existing alcohol-related brain imaging studies have focused on problematic alcohol consumers or those with AUD. The limited number of studies in non-AUD individuals have not explored the effects of a brief period of alcohol abstinence. Given the known rewarding properties of alcohol [27] and the aversive effects of abstinence [28,29], it is important to assess if everyday drinkers exhibit distinct neural responses during abstinence, compared to periods of typical consumption. In the current study, the relationship of PB_RSA_ to brain activity during abstinence was assessed using functional imaging. It is conceptually relevant to consider potential interactions between PB_RSA_ and brain state changes, as PB_RSA_ decreases with increases in threat to the organism. Alcohol craving, especially during abstinence, is highly associated with relapse in individuals recovering from AUD, and has been associated with distinct patterns of brain activity [30] and connectivity [31,32,33]. Chronic alcohol use is associated with reductions in grey and white matter volumes, with additional disruption to white matter tracts, neurotransmitter systems, and glucose metabolism [30]. When examining whole brain functional connectivity using graph theory metrics, few global differences were found between AUD patients and healthy controls. However, in males at high-risk of developing AUD, subnetworks (such as the attention network, executive control network, salience network, and default mode network) show a general expansion [32], marked by decreased clustering, small-worldness, and local efficiency [31]. In alcohol dependent inpatients, a negative relationship exists between average clustering coefficient and severity of alcohol use, and between dependence duration and global efficiency and clustering [33]. However, there is a lack of knowledge surrounding brain responses to temporary cessation of alcohol consumption when also accounting for functioning of the ANS. Combining these assessments of central and peripheral nervous system function has the potential to illuminate the broader underlying physiological consequences of alcohol abstinence. In an effort to test for these potential abstinence-driven changes in brain state, this study analyzes whole brain functional networks to achieve a comprehensive examination of the interaction of brain regions at a systematic level [34], rather than using activation analyses or connectivity measures from an a priori defined region of interest.

To best capture the mechanistic relationships between the central and peripheral nervous system, this study recruited everyday drinkers without behavioral problems associated with their consumption patterns. This allowed for comparison within individual participants, tracking brain network changes in response to their normal drinking patterns and to a brief period of imposed abstinence. The analyses performed focused primarily on clustering coefficient and global efficiency as network features that capture regional specificity and distributed processing, respectively. We hypothesized that changes in brain network topology between normal drinking and abstained states would be significantly associated with resting PB_RSA_. Specifically, individuals with higher resting PB_RSA_ would exhibit greater topological changes between the normal and abstained states, consistent with greater cognitive and emotional flexibility associated with higher resting PB_RSA._

## 2. Materials and Methods

### 2.1. Study Overview

The data used in this analysis come from a multi-part study examining neurobiological variables in everyday alcohol consumers. Previous manuscripts published from this same dataset examine patterns of craving and stress across the day and how RSA moderates patterns of craving. The drinking population studied in this protocol consisted of healthy adults whose lifestyle includes routine alcohol consumption above low risk levels [35] with infrequent binging episodes [36]. The study protocol consisted of a baseline visit and two magnetic resonance imaging (MRI) visits. After passing an initial telephone screening, a baseline visit was conducted to complete informed consent, ensure eligibility, administer self-report questionnaires, and collect cardiac functioning data. The MRI visits included self-report questionnaire administration and functional MRI (fMRI) scans. One visit occurred after three consecutive days of each participant’s normal drinking routine, and the other followed three consecutive days of imposed abstinence. This study implemented three-day experimental periods to achieve physiological consequences of abstinence in everyday drinkers without causing excess participant burden. Although the participants selected for this study were frequent drinkers, a single day of abstinence could be a common experience and subsequently not cause biological or psychological stress. A period of abstinence longer than three days was considered inconvenient and unpleasant to participants and was therefore a complication to recruitment and retention. The scanning order (normal versus abstained) was randomized with a crossover design, with a minimum of six days between scans. This report focuses on the relationship between resting state fMRI data (collected following abstinence and normal drinking) with resting RSA calculated from heart rate recordings from the baseline visit.

### 2.2. Participants

Thirty-four everyday drinkers were recruited from the community using various advertisement techniques including posted flyers, mailers, and inter/intranet postings. The final sample included 29 participants (13 males) after excluding for missing data (4 RSA, 1 fMRI). Enrollment criteria included adults aged 24–60, alcohol consumption on an average of ≥50% of days, and an average daily consumption of 1–3 drinks/day for females and 2–4 drinks/day for males. Drinking patterns were collected with the Timeline Followback (TLFB), a well-established retrospective measure of alcohol consumption [37]. Exclusion criteria included current or historical clinical AUD diagnosis, binge drinking [35,36] more than 3 occasions/month, a history of severe medical conditions stabilized for <2 months, a score of ≥20 on a depression inventory [38], history of neurological disease, consumption of >500 mg of caffeine per day, smoking >1.5 packs of cigarettes per day, or a positive urine drug screening (detecting for methamphetamine, cocaine, marijuana, amphetamines, opiates, and benzodiazepines). Due to the association between body mass index (BMI) and blood-alcohol concentration (BCA), BMI was restricted to a range of 18.5–39 [39]. Due to the MRI protocol, participants had to be right-handed, not claustrophobic, and have no contraindications to MRI.

### 2.3. Heart Rate Assessment and Data Processing for RSA Calculation

The primary research question raised in this study examined resting levels of PB_RSA_, a phenotypic index of vagal health. As such, PB_RSA_ was calculated from heart rate data collected at baseline, before the study-imposed abstinence period. PB_RSA_ was then analyzed in conjunction with functional MRI data collected following the normal drinking and abstinence experimental periods. An electrocardiogram (ECG) was collected via a Biopac MP150 system during the baseline visit. A three-electrode configuration was used in conjunction with a pulse oximeter. Participants were instructed to refrain from speaking and remain still throughout data collection. Heart rate was recorded for 5 min while participants were seated comfortably. CardioEdit software (Brain-Body Center, University of Illinois at Chicago, 2007) was used to visually inspect and edit off-line heart rate data. CardioBatch Plus software (Brain-Body Center for Psychophysiology and Bioengineering, University of North Carolina at Chapel Hill, 2016) was used to calculate heart rate and PB_RSA_ from the ECG data consistent with procedures developed by Porges [40]. This baseline calculation was used because not all intercorrelated parasympathetic metrics are equivalent, and RSA metrics do not need to be statistically adjusted for ventilatory parameters to accurately estimate CVT [41].

These methods have been documented to extract a single amplitude of PB_RSA_ as a valid index of HRV [41]. CardioBatch Plus quantified the amplitude of PB_RSA_ using age-specific parameters that are sensitive to the maturational shifts in the frequency of spontaneous breathing. The method, when applied to adults, includes: (1) timing sequential R-R intervals to the nearest millisecond; (2) producing time-based data by resampling the sequential R-R intervals into 500 ms intervals; (3) detrending the time-based series with a 21-point cubic moving polynomial stepped through the data to create a smoothed template, then subtracting the template from the original time-based series to generate a detrended residual series; (4) bandpass filtering the detrended time series to extract the variance in the heart period pattern associated with spontaneous breathing in adults (0.12–0.40 Hz); and (5) transforming the variance estimates with a natural logarithm to normalize the distribution of PB_RSA_ estimates [42]. These procedures [40] are statistically equivalent to frequency domain methods (i.e., spectral analyses) for the calculation of the amplitude of RSA when heart period data are stationary [23]. This calculation method is not moderated by respiration rate or amplitude, and is equivalent to applying spectral methods following our filtering technique [41]. PB_RSA_ was quantified during each sequential 30 s epoch and the averages within each condition were used in the data analysis.

### 2.4. Brain Imaging and Functional Brain Network Analysis

#### 2.4.1. MRI Study Visits

At the beginning of each MRI session, participants completed surveys regarding any changes to their typical routine, and probing alcohol craving [43], stress [44], anxiety [45], and mindfulness [46,47]. Following the abstinence experimental period, participants also completed the Clinical Institute Withdrawal Assessment for Alcohol-revised (CIWA-Ar) [48] to ensure no participants experienced withdrawal symptoms. These scales were administered to help determine any differences between these participants’ emotional and physical states across the two experimental periods. No significant differences were found between the drinking states on any of these measures (Supplemental Table S4). These scores were not included in statistical modeling with brain networks as they may be causal intermediaries.

#### 2.4.2. Image Collection

MRI data was obtained on a 3T Siemens Skyra scanner equipped with a 32-channel head coil, a rear projection screen, and MRI compatible headphones. The imaging protocol consisted of a T1-weighted structural scan followed by a 6 min blood oxygen level dependent (BOLD)-weighted resting state scan. During the resting state scan, participants were instructed to focus on a fixation cross projected on the rear projection screen. High resolution (1 mm isotropic) T1-weighted structural scans were acquired in the sagittal plane using a single-shot 3D MPRAGE GRAPPA2 sequence (repetition time (TR) = 2.3 s, echo time (TE) = 2.99 ms, 192 slices). The resting state BOLD-weighted image sequences were acquired in the transverse plane using an echo-planar imaging sequence (3.5 mm × 3.5 mm × 5 mm resolution, acquisition time = 6 min, TR = 2.0 s, TE = 25 ms, flip angle = 75°, 35 slices per volume, 177 volumes).

#### 2.4.3. Image Processing and Network Generation

The first 20 s (10 image volumes) were discarded to allow the signal to achieve equilibrium. Initial steps for image processing were performed using SPM12 software (www.fil.ion.ucl.ac.uk/spm/, accessed on 12 October 2018). The functional images were slice time corrected and realigned to the first image of the series. Preprocessing for the structural image consisted of skull removal with the remaining image segmented into grey matter, white matter, and cerebrospinal fluid (CSF) maps using a unified segmentation algorithm [49]. The structural image was warped to the Colin template [50] using Advance Normalization Tools (ANTS) [51]. The resulting inverse warp deformation map was applied to the Shen functional atlas [52], warping the atlas to each subject’s original (native space) anatomical image. The atlas was then co-registered and resliced to match functional data. The Shen atlas contains 268 functionally defined regions, specifically defined for brain network analyses [52].

Physiological noise and low frequency drift were reduced by regressing out the mean signals for grey matter, white matter, and CSF and applying band-pass filtering (0.009–0.08 Hz) [53]. Motion correction was performed to eliminate scan volumes with excessive frame-wise displacement and BOLD signal change [54]. Each participant’s native space atlas was used to extract the mean time series for each region of the Shen atlas. It is of note that all brain networks examined in this study were created and analyzed in each participant’s native space to limit manipulation and interpolation of the fMRI time series. These functional atlas time series data were used to generate functional brain networks by performing node-by-node Pearson’s correlations using the WFU_MMNET toolbox [55]. Statistical analysis focused on unthresholded positive correlation matrices with negative correlation values set to 0. The mixed-model framework used in this brain network analysis relies on graph theory metrics that cannot accommodate negative correlations [56].

#### 2.4.4. Statistical Analysis and Mixed-Effects Modeling Framework

Full details regarding the methodology used for statistical modeling are included in the Supplementary Materials.

A mixed-effects regression was used to assess the relationship between whole brain network connectivity, participants’ drinking state, PB_RSA_ as a continuous measure of CVT, and possible confounding variables [56,57,58]. This framework is able to account for brain network correlations within each participant (within subject variables) across two states (following normal drinking and abstinence), and allows the inclusion of network and non-network variables (between subject variables) in the model. Significant results were determined by a critical *p*-value <0.05, with *p*-values adjusted for multiple comparisons using the adaptive False Discovery Rate procedures detailed by Benjamini and Hochberg [59]. Analyses were conducting using the WFU_MMNET toolbox [55], Matlab (R2016), and SAS v9.4 software.

The goal of this analysis was to determine if brain network differences observed across drinking states were driven by HRV. To test this relationship, the model allowed for the comparison of these variables with the strength of network connections, or the similarity of the data recorded from each brain region. More simply, this regression model determined how much each of these variables affected the similarity of data collected from different brain regions, indicating how connected they are as nodes in the whole brain functional network. The variables included average clustering coefficient (local segregation), average global efficiency (global integration), difference in degree (number of connections) between each nodal pair, and overall network modularity (the extent to which the network subdivides into densely interconnected communities that are scarcely connected to the rest of the network) [60] as well as age, sex, and BMI [39,61,62]. These non-network variables were included as covariates in the model to control for any associations with network organization.

## 3. Results

The mixed-model framework tested the hypothesis that the effects of drinking state on brain network topology are resting levels of PB_RSA_. The results section highlights key findings with the full model results being available in the Supplemental Materials.

Descriptive statistics related to participants’ demographic characteristics and variables included in the statistical model are listed in Table 1. A total of 29 participants (13 males) completed the full study protocol. Participants averaged an age of 38.8 years with an average BMI of 24.8. Of the full sample, 6.89% of participants identified as African American or black, 3.45% identified as Asian, and 89.66% identified as white. The participants had been consuming alcohol for an average of 18.9 years and consumed an average of 2.3 drinks on an average of 81.2% of days in the last three months (as determined with the TLFB). There were no significant differences between males and females for any demographic variable, including our alcohol consumption variables. Age, sex, and BMI were not significant predictors of brain network structure.

**Figure 1:**
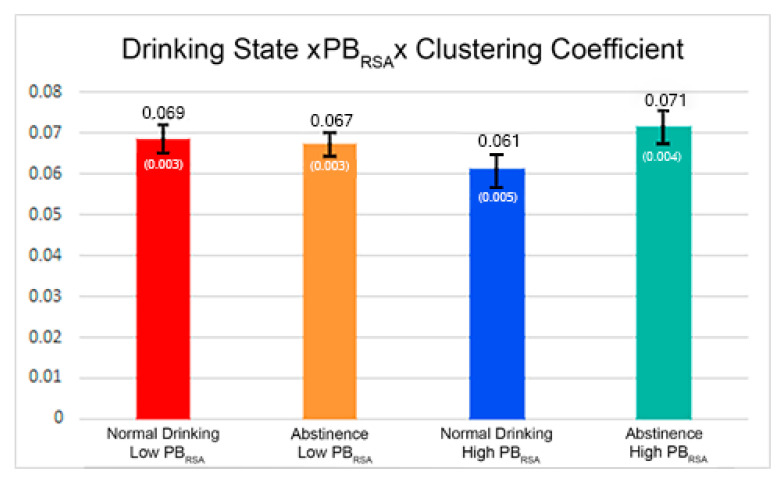
The bars capture the slope of the relationship between clustering coefficient and connection strength, across drinking states and as PB_RSA_ increases. Numbers above the bar are slope values, and numbers in parentheses are standard error. At lower RSA values, there is minimal difference in the relationship between drinking states, whereas at higher PB_RSA_ values, there is a much larger change in the slope of the relationship between clustering and strength.

The primary finding from the brain network analysis was a significant interaction between the connection strength, clustering coefficient, drinking state, and PB_RSA_ (β = 0.01186, *p* = 0.0004), as shown in Table 2. This finding indicates that the magnitude of the strength-clustering relationship differs more across drinking states as PB_RSA_ values increase. Thus, higher PB_RSA_ values are associated with networks that have the strongest connections between highly clustered nodes during abstinence. At lower PB_RSA_ values, only a small difference was observed in the clustering-strength relationship across drinking states, with weak network connections between highly clustered nodes during abstinence. These differences are meaningful as stronger network connections support synchronization of neural signals across regions, leading to efficient information sharing between clusters. The slope of the regression captures the magnitude of the strength-clustering relationships and is shown for each state at higher and lower PB_RSA_ values in Figure 1.

**Figure 2:**
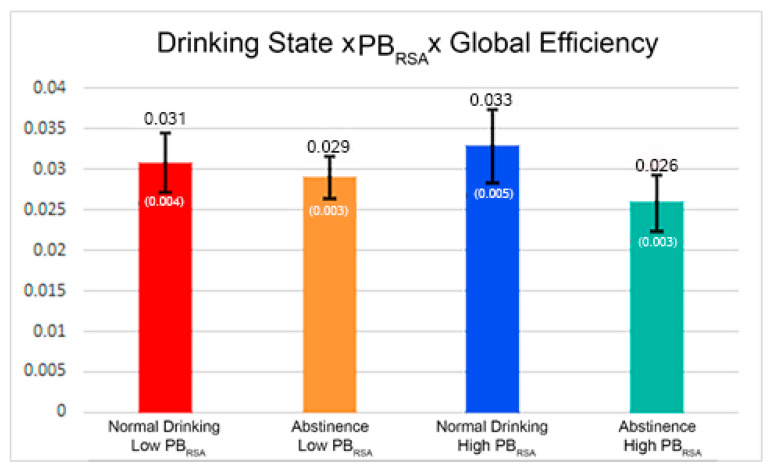
(Trending only). The bars capture the slope of the relationship between global efficiency and connection strength, across drinking states and as PB_RSA_ increases. Numbers above the bar are slope values, and numbers in parenthesis are standard error. At both the upper and lower bounds of PB_RSA_, the relationship between global efficiency and strength was steeper during normal drinking. At lower PB_RSA_ values, the slope decreased minimally during abstinence, whereas at higher PB_RSA_ values, the slope decreased to a more significant degree following abstinence.

A second connection strength relationship was found with global efficiency, drinking state, and PB_RSA_ but this finding only trended toward significance (β = −0.00518, *p* = 0.1087). This statistical trend is presented here because it may be critical for understanding the mechanistic relationships between PB_RSA_ and brain networks. Across all observed PB_RSA_ values, highly globally efficient nodes were more strongly connected during abstinence. The slope of this relationship was steeper at lower PB_RSA_ values, indicating the strength of connections between highly globally efficient nodes increases more rapidly at lower PB_RSA_ values than at higher PB_RSA_ values. This would result in a higher probability of synchronization between highly globally efficient nodes, increasing the distribution of information globally. The slope of these relationships can be observed in Figure 2. Although this relationship did not reach significance, the topology differences observed across drinking states were large, and the directionality of the findings conceptually fit with the highly significant clustering findings. One would expect that as clustering increases, global efficiency should decrease, and vice versa, as a tradeoff should be expected between local segregation and global communication in a network.

Table 2 shows these key results from the mixed-model, and full results are included in the Supplemental Material. In addition to these significant 3-way interactions, there was also a significant main effect of drinking state, meaning the strength of connections across the network were stronger during normal drinking (β = 1.2340, *p* < 0.0001). We also observed a significant main effect of PB_RSA_, indicating that as PB_RSA_ increases network connectivity strength decreases (β = −0.00994, *p* = 0.0220). Additionally, there was an interaction between drinking state and PB_RSA_ that did not reach statistical significance but exhibited a notable positive trend (β = 0.01180, *p* = 0.0553). Clustering coefficients (β = 0.06825, *p* < 0.0001) and global efficiency (β = 0.02923, *p* < 0.0001) were both significant positive predictors of connection strength. Finally, there was an interaction of PB_RSA_ with clustering (β = −0.00532, *p* = 0.0004) but not with global efficiency.

## 4. Discussion

This study examined how HRV relates to brain network topology changes between periods of normal drinking and alcohol abstinence in everyday drinkers. The main study hypothesis was supported by the results found in this study: differences in the relationship between the strength of network connections and clustering coefficients across drinking states was mediated by PB_RSA_ measured at rest. These results showed increasing connection strength between highly clustered nodes during abstinence as PB_RSA_ increases, and these network changes suggest a greater range of possible topological configurations at high PB_RSA_ values. A thorough understanding of the biological effects underlying alcohol abstinence will help lay a foundation of alcohol research focused on development of clinical treatment and prevention strategies related to alcohol consumption.

To fully understand the implications of these findings, we must remember what the statistical network model is evaluating. In a traditional functional brain network built with Pearson’s correlations, as a network feature such as clustering coefficient or global efficiency between two nodes increases, the strength of the edge connecting those nodes should also increase [56]. The strength of connection between nodes, or how similar the fMRI data collected from each brain region is, can have important implications for overall network topology and resultant information processing. The stronger an edge connecting two nodes, the more likely those nodes will synchronize and share information effectively [63], which is important because synchronization between brain regions is believed to be an essential component of neural processing [64]. However, weak connections are not irrelevant; that is, although two nodes connected by a weak link are not as likely to synchronize, they can be a bridge for one node to introduce novel information to a group of nodes to which it is not strongly connected [65,66]. Our results showed that network organization was influenced by drinking state more as individuals’ PB_RSA_ values increased. Among those with higher PB_RSA_ values following abstinence, we found strong connections between highly clustered nodes and weaker connections between globally efficient nodes. However, during normal drinking, we would expect weaker connections between highly clustered nodes. At lower PB_RSA_ values, the edges connecting highly globally efficient nodes would be expected to be weaker during abstinence. When considering combined effects, the clustering and global efficiency findings suggest that as PB_RSA_ values increase, the network takes on a more lattice-like structure during abstinence, and a more small-world topology during normal drinking [67], with a more intermediate network structure at lower PB_RSA_ values. These findings have substantial implications for the exchange of information across the whole brain.

As biomedical sciences take a broader approach to examining alcohol disorders, treatment, and prevention, there is a growing relevance of novel translational biomarkers. HRV has potential as a translational biomarker, in relation to alcohol consumption, and more broadly with emotional or cognitive deficits, as dysfunction in HRV is observed in many psychiatric disorders [68,69,70]. Low HRV is a cardiovascular risk factor, commonly caused by persistent activation of the sympathetic nervous system [71]. Optimal heart rate should change throughout the day in response to environmental changes resulting in excitement, anger, anxiety, or other emotional experiences. Low HRV may reflect heart rate pattern that inefficiently does not make these adaptive adjustments or is “stuck” within a narrow band. While higher HRV is generally believed to be beneficial, there are cases in which higher parasympathetic tone is associated with increased cardiac risk, such as in congenital long QT syndrome (LQTS) (a cardiac arrhythmia syndrome and a leading cause of sudden death in youth). With impaired QT shortening, higher HRV values allowing for sudden changes in heart rate could result in the initiation of ventricular tachycardia-fibrillation [72,73]. LQTS mutation carriers have been shown to have lower heart rate and lower baroreflex sensitivity, indicating low parasympathetic tone may potentially be a marker of their autonomic dysfunction [74,75,76]. Low HRV itself is not usually a disorder, but it can be a sign that something is amiss. For example, low HRV is common in major depressive disorder and can lead to fatal dysrhythmias. With depression, the belief is that sympathetic hyperactivity reflected in lowering of HRV, also drives the experiences of anxiety, agitation, and dysphoria [71]. Additionally, anger, aggression, fatigue, and vigor associated with depressive episodes in otherwise healthy young individuals are all associated with the dysregulation of the parasympathetic component of HRV [77]. Balance of autonomic nervous system functioning is also correlated with cognitive performance—dysfunctions are known to precede cognitive impairment [78]. A lowering of HRV observed with decreased parasympathetic activity is associated with worse performance in all cognitive domains, and is considered a potential early biomarker of cognitive impairments in populations without dementia or stroke [78]. Low RSA values are observed in conjunction with aging, chronic stress, and medical and psychiatric disorders, and have predictive validity for all-cause mortality [68]. Higher RSA values are associated with a greater dynamic range in parasympathetic function and have been found to be physiologically advantageous [23,79]. This wide foundation of research demonstrates the relationship between RSA and pathology, and a growing body of literature has begun connecting alcohol use with autonomic nervous system disruptions [13,14,15,21,68,80]. The current study drew novel connections between HRV and functional brain networks in everyday alcohol consumers, observing a significant relationship between shifts in brain network topology and HRV.

This study is not without weaknesses that should be recognized. While the interaction involving clustering was highly significant, the interaction with global efficiency did not reach statistical significance. Nevertheless, the global efficiency findings fit conceptually with the clustering findings, and future work is needed to replicate these findings in order to determine the implications of the global efficiency finding. The relatively small sample size in the current study could have contributed to the lack of significance. It should also be noted that the small sample size may limit the generalizability of the findings. However, robust significant effects were found despite the small sample size. Another potential limitation was the timing of MRI scanning: all fMRI data used in this protocol was collected between 8 am and 12 pm on the fourth day of each normal drinking/abstinence week. Previous analyses in this population have shown limited craving for alcohol, even during abstinence, until late afternoon [17,29], meaning scans collected during the morning may not adequately capture abstinence effects in this population. Studies that have aligned the time of brain imaging with individual participants’ peak levels of craving are currently underway. From this study alone, it is unclear where these changes might be manifesting across the whole brain, as the methods used only allowed for examination of differences at a macroscopic level. Therefore, future studies should expand on these findings by examining the locations of these PB_RSA_ dependent network changes. Finally, because this study was specifically interested in a resting PB_RSA_ phenotype and therefore used a baseline measure to associate with brain network topology, our study is not able to assess the effects of abstinence on PB_RSA_. However, this is an important question that warrants further study in the future.

## 5. Conclusions

Regular alcohol consumption is common practice in our world today. The largest study of alcohol use to-date found that at least 60% of the US population consumes 1–3 alcoholic beverages per day [1]. The goal of the present study was to examine changes in functional brain network structure driven by autonomic nervous system function observed during a period of alcohol abstinence, but as knowledge is gained regarding these neurobiological underpinnings, there is potential for significant impact on research focused on non-AUD drinkers or AUD sufferers. Ultimately, understanding clinical issues related to alcohol consumption relies on our understanding of the basic mechanisms underlying brain-behavior relationships. An understanding of the interaction between ANS and central neural adaptations surrounding alcohol abstinence is essential to understand more clinically based concerns and may lead to the discovery of signatures for potential diagnostic, prognostic, and treatment targets in studies with a clinical focus. Overall, this study documented changes in functional brain network topology across drinking states dependent on ANS function. Results showed that while brain networks do differ across drinking states in risky drinkers, the change is primarily driven by HRV, measured via PB_RSA_.

## Figures and Tables

**Table 1:** Sample demographics. Listed as Mean (Standard Deviation) or Frequency (Percentage), [range]. There were no significant differences between males and females in any variable.

Variable	Overall (*n* = 29)	Male (*n* = 13)	Female (*n* = 16)
Age	38.8 (10.9) [24–60]	36.6 (6.6) [24–46]	40.5 (13.3) [24–60]
BMI	24.8 (3.8) [18.9–39]	25.6 (3.5) [20.4–33.4]	24.2 (4.0) [18.9–39]
Race *n* (%)			
African American or Black	2 (6.89%)	2 (15.38%)	0
Asian	1 (3.45%)	1 (8.34%)	0
White	26 (89.66%)	10 (83.34%)	16 (100%)
Alcohol Use			
Total Years Drinking	18.9 (10.8) [4–40]	17.3 (7.2) [6–30]	20.1 (13.1) [4–40]
Timeline Followback ^1^			
Percent of Days that were Drinking Days	81.2% (16.0) [55–100%]	78.6% (16.4) [55.21–100%]	83.4% (15.8) [55–100%]
Average Drinks Consumed on Drinking Days	2.3 (0.73) [1.02–5.43]	2.4 (0.26) [2.02–2.78]	2.3 (0.96) [1.02–3.88]
Cardiac Vagal Tone			
PB_RSA_	5.8 (1.69) [0.44–7.90]	6.0 (0.93) [4.44–7.49]	5.6 (2.13) [0.44–7.90]

^1^ Drinking pattern during the previous 90 days.

**Table 2:** Relevant mixed-model strength results. Full model results are available in the Supplementary Material.

Effect	Estimate	Standard Error	Adaptive FDR *p*-Value
Intercept	0.2340	0.004659	<0.0001
Drinking State	−0.01502	0.005259	0.0133
PB_RSA_-rest	−0.00994	0.004340	0.0616
Drinking State*PB_RSA_-rest	0.01180	0.006157	0.1106
Clustering Coefficient	0.06825	0.002450	<0.0001
Global Efficiency	0.02923	0.002528	<0.0001
Clustering Coefficient*Drinking State	0.001887	0.003163	0.5509
Global Efficiency*Drinking State	−0.00456	0.003046	0.1981
Clustering Coefficient*PB_RSA_-rest	−0.00532	0.002651	0.0967
Global Efficiency*PB_RSA_-rest	0.000041	0.002713	0.9879
Clustering Coefficient*Drinking State*PB_RSA_-rest	0.01186	0.003357	0.0014
Global Efficiency*Drinking State*PB_RSA_-rest	−0.00518	0.003226	0.1749
Age	−0.00116	0.003233	0.7187
Sex	−0.00012	0.005968	0.9843
BMI	−0.00348	0.003519	0.3786

## Data Availability

The data analyzed in this study is not currently publically available. However, the software used for analysis are available at https://www.nitrc.org/projects/wfu_mmnet.

## Supplementary Materials

The following are available online at https://www.mdpi.com/2076-3425/11/6/817/s1, Supp. Methods; Supp. Results; Supp. Table S1: Relevant mixed-model strength results; Table S2: Full mixed-effects model results-strength; Table S3: Full mixed-effects model results-probability; Table S4: Average survey responses; Figure S1: Drinking State × PBRSA-rest × Clustering Coefficient × Connection Strength; Figure S2: Drinking State × PB_RSA_-rest × Global Efficiency × Connection Strength; Figure S3: PB_RSA_-rest × PB_RSA_-react × Clustering Coefficient × Connection Strength; Figure S4: PB_RSA_-rest × PB_RSA_-react × Global Efficiency × Connection Strength.
