# Correction: Peterson et al. Influence of Heart Rate Variability on Abstinence-Related Changes in Brain State in Everyday Drinkers. *Brain Sci.* 2021, *11*, 817

**DOI:** 10.3390/brainsci12050624

**Published:** 2022-05-10

**Authors:** Hope Peterson, Rhiannon E. Mayhugh, Mohsen Bahrami, Walter Jack Rejeski, Sean L. Simpson, Keri Heilman, Stephen W. Porges, Paul J. Laurienti

**Affiliations:** 1 Laboratory for Complex Brain Networks, Wake Forest University Health Sciences, Winston-Salem, NC 27104, USA; mbahrami@wakehealth.edu (M.B.); rejeski@wfu.edu (W.J.R.); slsimpso@wakehealth.edu (S.L.S.); plaurien@wakehealth.edu (P.J.L.); 2 Graduate Program, Wake Forest School of Medicine, Neuroscience, Winston-Salem, NC 27101, USA; 3 Professional Development and Career Office, Johns Hopkins University School of Medicine, Baltimore, MD 21287, USA; rhiannon.mayhugh@gmail.com; 4 Health and Exercise Science, Wake Forest University, Winston-Salem, NC 27109, USA; 5 Wake Forest School of Medicine, Biostatistics and Data Science, Winston-Salem, NC 27101, USA; 6 Department of Psychiatry, University of North Carolina School of Medicine, Chapel Hill, NC 27514, USA; heilmank@sbcglobal.net (K.H.); sporges@gmail.com (S.W.P.); 7 Department of Psychiatry, University of Illinois, Chicago, IL 60612, USA; 8 Wake Forest School of Medicine, Radiology, Winston-Salem, NC 27101, USA

## Error in Table

In the original article [1], there was a mistake in Table 2 as published. The model intercept was misidentified as the covariate “Drinking State”, and uncorrected *p*-values were listed rather than Adaptive FDR-corrected *p*-values, as stated in the text. The corrected Table 2 appears below. 

## Error in Supplemental Table

In the original article, there was a mistake in Tables S2 and Table S3 as published. Uncorrected *p*-values were listed rather than Adaptive FDR-corrected *p*-values, as stated in the text. The corrected Tables S2 and Table S3 appear below. 

The authors apologize for any inconvenience caused and state that the scientific conclusions are unaffected. The original article has been updated.

## Figures and Tables

**Table 2:** Relevant mixed-model strength results. Full model results are available in the Supplementary Material.

Effect	Estimate	Standard Error	Adaptive FDR *p*-Value
Intercept	0.2340	0.004659	<0.0001
Drinking State	−0.01502	0.005259	0.0133
PB_RSA_-rest	−0.00994	0.004340	0.0616
Drinking State*PB_RSA_-rest	0.01180	0.006157	0.1106
Clustering Coefficient	0.06825	0.002450	<0.0001
Global Efficiency	0.02923	0.002528	<0.0001
Clustering Coefficient*Drinking State	0.001887	0.003163	0.5509
Global Efficiency*Drinking State	−0.00456	0.003046	0.1981
Clustering Coefficient*PB_RSA_-rest	−0.00532	0.002651	0.0967
Global Efficiency*PB_RSA_-rest	0.000041	0.002713	0.9879
Clustering Coefficient*Drinking State*PB_RSA_-rest	0.01186	0.003357	0.0014
Global Efficiency*Drinking State*PB_RSA_-rest	−0.00518	0.003226	0.1749
Age	−0.00116	0.003233	0.7187
Sex	−0.00012	0.005968	0.9843
BMI	−0.00348	0.003519	0.3786

**Table S2:** Full mixed-effects model results—strength.

Effect	Estimate	Standard Error	Adaptive FDR *p*-Values
Intercept	0.2340	0.004659	<0.0001
Drinking State	−0.01502	0.005259	0.0133
PB_RSA_-rest	−0.00994	0.004340	0.0616
PB_RSA_-react	0.007051	0.004156	0.1676
Clustering Coefficient	0.06825	0.002450	<0.0001
Global Efficiency	0.02923	0.002528	<0.0001
Degree	−0.04430	0.001256	<0.0001
Modularity	−0.01518	0.003053	<0.0001
Age	−0.00116	0.003233	0.7187
Sex	−0.00012	0.005968	0.9843
BMI	−0.00348	0.003519	0.3786
Distance	−0.05132	0.001100	<0.0001
Distance^2^	0.02890	0.000537	<0.0001
Clustering Coefficient*PB_RSA_-rest*PB_RSA_-react	−0.00223	0.001092	0.0967
Global Efficiency*PB_RSA_-rest*PB_RSA_-react	0.001999	0.000993	0.0967
Degree*PB_RSA_-rest*PB_RSA_-react	−0.00051	0.000561	0.3874
Modularity*PB_RSA_-rest*PB_RSA_-react	−0.00570	0.004092	0.2291
Clustering Coefficient*Drinking State*PB_RSA_-rest	0.01186	0.003357	0.0014
Global Efficiency*Drinking State*PB_RSA_-rest	−0.00518	0.003226	0.1749
Degree*Drinking State*PB_RSA_-rest	−0.00120	0.001734	0.4897
Modularity*Drinking State*PB_RSA_-rest	−0.00138	0.007352	0.8511
Clustering Coefficient*Drinking State*PB_RSA_-react	−0.00329	0.003343	0.3786
Global Efficiency*Drinking State*PB_RSA_-react	0.002779	0.003215	0.3874
Degree*Drinking State*PB_RSA_-react	0.001632	0.001730	0.3871
Modularity*Drinking State*PB_RSA_-react	−0.01339	0.008060	0.1692
Clustering Coefficient*PB_RSA_-rest	−0.00532	0.002651	0.0967
Global Efficiency*PB_RSA_-rest	0.000041	0.002713	0.9879
Degree*PB_RSA_-rest	−0.00049	0.001351	0.7183
Modularity*PB_RSA_-rest	0.003155	0.003607	0.3874
Clustering Coefficient*PB_RSA_-react	0.000271	0.002731	0.9209
Global Efficiency*PB_RSA_-react	0.001526	0.002783	0.5835
Degree*PB_RSA_-react	−0.00154	0.001399	0.3530
Modularity*PB_RSA_-react	0.006400	0.005891	0.3530
Clustering Coefficient*Drinking State	0.001887	0.003163	0.5509
Global Efficiency*Drinking State	−0.00456	0.003046	0.1981
Degree*Drinking State	0.000871	0.001643	0.5961
Modularity*Drinking State	−0.00044	0.005495	0.9367
Drinking State*PB_RSA_-rest	0.01180	0.006157	0.1106
Drinking State*PB_RSA_-react	−0.00886	0.005583	0.1749
PB_RSA_-rest*PB_RSA_-react	−0.00021	0.002646	0.9366

**Table S3:** Full mixed-effects model results—probability.

Effect	Estimate	Standard Error	Adaptive FDR *p*-Values
Intercept	0.1803	0.03466	<0.0001
Drinking State	−0.00310	0.04010	0.9383
PB_RSA_-rest	0.01808	0.03196	0.7998
PB_RSA_-react	0.01719	0.03062	0.7998
Clustering Coefficient	0.3214	0.03946	<0.0001
Global Efficiency	−0.3051	0.03295	<0.0001
Degree	0.1480	0.02142	<0.0001
Modularity	−0.02214	0.02248	0.7304
Age	−0.00490	0.02444	0.8502
Sex	0.05785	0.04534	0.6965
BMI	−0.02645	0.02663	0.7304
Distance	0.3597	0.01688	<0.0001
Distance^2^	−0.1346	0.007011	<0.0001
Clustering Coefficient*PB_RSA_-rest*PB_RSA_-react	−0.01727	0.01506	0.6965
Global Efficiency*PB_RSA_-rest*PB_RSA_-react	0.002236	0.01408	0.8738
Degree*PB_RSA_-rest*PB_RSA_-react	0.007827	0.01016	0.7998
Modularity*PB_RSA_-rest*PB_RSA_-react	0.02910	0.03130	0.7466
Clustering Coefficient*Drinking State*PB_RSA_-rest	0.06126	0.04975	0.6965
Global Efficiency*Drinking State*PB_RSA_-rest	−0.02835	0.04411	0.7998
Degree*Drinking State*PB_RSA_-rest	−0.03307	0.03111	0.7304
Modularity*Drinking State*PB_RSA_-rest	−0.01012	0.05689	0.8588
Clustering Coefficient*Drinking State*PB_RSA_-react	0.01006	0.04971	0.8502
Global Efficiency*Drinking State*PB_RSA_-react	−0.00360	0.04409	0.9349
Degree*Drinking State*PB_RSA_-react	−0.01732	0.03110	0.7998
Modularity*Drinking State*PB_RSA_-react	−0.02415	0.06135	0.8502
Clustering Coefficient*PB_RSA_-rest	0.002919	0.04219	0.9448
Global Efficiency*PB_RSA_-rest	−0.01796	0.03530	0.8145
Degree*PB_RSA_-rest	0.04652	0.02302	0.2224
Modularity*PB_RSA_-rest	−0.03170	0.02644	0.6965
Clustering Coefficient*PB_RSA_-react	−0.02654	0.04336	0.7998
Global Efficiency*PB_RSA_-react	0.02398	0.03655	0.7998
Degree*PB_RSA_-react	0.005727	0.02403	0.8502
Modularity*PB_RSA_-react	0.008263	0.04331	0.8502
Clustering Coefficient*Drinking State	−0.05454	0.04713	0.6965
Global Efficiency*Drinking State	0.04795	0.04181	0.6965
Degree*Drinking State	0.01711	0.02950	0.7998
Modularity*Drinking State	−0.01085	0.04249	0.8502
Drinking State*PB_RSA_-rest	−0.03603	0.04698	0.7998
Drinking State*PB_RSA_-react	−0.01413	0.04272	0.8502
PB_RSA_-rest*PB_RSA_-react	0.007968	0.01971	0.8502
