# Supplementary material for: Influence of Heart Rate Variability on Abstinence-Related Changes in Brain State in Everyday Drinkers
Source: Brain Sci. 2021 Jun 20;11(6):817. doi: 10.3390/brainsci11060817 (PMC8235786; doi:10.3390/brainsci11060817)
Supplement: Supplementary file 1 [file brainsci-11-00817-s001.zip › brainsci-1662960-supplementary.pdf]

brain sciences

MDPI

Supplementary Material

1. Supplementary Methods

1.1 Heart Rate Assessment and Data Processing for PB_{RSA} Calculation

In addition to assessment of PB_{RSA} data collected while participants were seated (PB_{RSA}-rest), PB_{RSA} was also calculated on heart rate data collected following a postural shift (seated to standing; PB_{RSA}-react). The main outcomes for PB_{RSA}-react data were negative, and as such were excluded from discussion of primary results. Using the ECG 3-electrode set up, data was collected for 5 minutes while participants were seated comfortably, and for 2 minutes following the postural shift. CardioEdit and CardioBath Plus software were used to inspect the data and calculate heart rate as described in the paper methods. To calculate PB_{RSA}-react scores, the average 5 minute seated PB_{RSA} value (PB_{RSA}-rest) was subtracted from the PB_{RSA} recorded during the standing 2 minutes.

1.2 Statistical Analysis and Mixed-Effects Modeling Framework

A mixed-effects regression was used to assess the relationship between whole brain connectivity, participants' drinking state (normal vs. abstain), continuous measure of HRV (PB_{RSA}), and possible confounding variables [1, 2]. This framework was able to account for correlations between network connections within the individual participants and between the normal and abstained drinking states, as well as allow for the analysis of both network and non-network variables. This two-part regression models the *probability* of network connections (binary; presence vs. absence) and the *strength* of connections, if they exist. The multivariate regression captures the relationship between brain connections (probability and strength) as outcome (dependent) variables and network/non-network covariates as the independent variables. For all analyses, significant results were determined by a critical p-value < 0.05. All p-values were adjusted for multiple comparisons using the adaptive False Discovery Rate procedures detailed by Benjamini and Hochberg [3]. Analyses were conducted using the WFU_MMNET toolbox [4], Matlab (R2016b), and SAS v9.4 software.

Figures were generated from the resulting fits of the mixed effects regression model. For visualization purposes, the upper and lower bounds of the PB_{RSA} relationships were used in the regression equations in conjunction with the dichotomous drinking state variable to depict graphs. It should be remembered when interpreting these figures that they are intended for visualization, and that the statistical analyses are based on continuous functions that span the region between the upper and lower bounds.

1.3 Covariates

The primary variables of interest in this model were drinking state and HRV. Because fMRI time series data from both drinking states were included in the statistical model, a dichotomous variable was coded to differentiate the two conditions (normal drinking = 0, abstinence = 1). This allowed for the examination of the effects of drinking state between and within subjects, as well as the interaction of drinking state with network properties and other covariates. The complete model included interactions of all covariates and network features with both PB_{RSA}-rest and PB_{RSA}-react, but no 4-way interactions (i.e. probability/strength* PB_{RSA}-rest* PB_{RSA}-react*network feature) were significant, and were subsequently remove from the model to focus on significant 3-way interactions.

The network topology [5] from each individual participant was summarized with standard graph theory variables computed from each node pair, including average *clustering coefficient* (local specialization), average *global efficiency* (global integration), differences in *degree* (number of connections) between each nodal pair, and overall network *modularity* (the extent to which the network subdivides into densely interconnected communities that are scarcely connected to the rest of the network) [6]. Each of these summary variables was included as covariates in the model.

Age, sex, and BMI were included in the model due to previous associations with alcohol use and HRV [7-9]. The spatial Euclidean distance and square of the spatial distance between network nodes (brain regions) were also included as confounding variables to account for participants' inter/intra-hemispheric asymmetries [10]. These metrics were included as covariates in the model to control for any associations with network organization. Measures that capture variance in drinking behavior and alcohol exposure were entered in a secondary post hoc model as additional covariates. These included average number of drinks consumed per day when participants chose to drink, the percent of days that they reported drinking at least one serving of alcohol in the last three months (via the TLFB), and the total number of years they had been consuming alcohol.

2. Supplementary Results

2.1 Primary Findings

The two-part mixed-model test the hypothesis that the effects of drinking state on brain network topology are dependent on HRV, measured via PB_{RSA}. This model separately evaluated connection strength and probability. Results for the connection strength model are presented here, and results for the connection probability model did not achieve significance for the stated hypothesis, and there were no significant interactions between PB_{RSA}-react and drinking state. The full probability model and results for PB_{RSA}-react are presented in the following section, covering the full model findings.

Degree, modularity, clustering coefficient, and global efficiency were included in the model as network covariates. No meaningful relationships with drinking state were found with modularity or degree. All four network features network features remained in the full model, but are only presented in the full model findings. The full model also included age, sex, and BMI to control for confounding effects. None of the demographic variables were significantly associated with connection strength. To control for differences in long vs. short distance brain connections, the spatial distance and square of the spatial distance between brain regions were included in this model. Distance variables were significant (Spatial Distance $\beta = -0.05132$, $p < 0.0001$, Spatial Distance^2 $\beta = 0.02890$, $p < 0.0001$).

Two 3-way interactions were associated with the main study hypothesis. The most robust interaction from the connection strength model was between drinking state, PB_{RSA}-rest, and clustering ($\beta = 0.01186$, $p = 0.0004$). This 3-way interaction can be visualized by examining the slope of the relationship between connection strength and clustering across drinking state and the upper and lower bounds of PB_{RSA}-rest values (Supp. Fig. 1). At the upper bound of PB_{RSA}-rest values large differences were found in the relationship between connection strength and clustering coefficient across drinking states, while at the lower bound only small differences were present. To understand these differences in the relationship between clustering and connection strength, we examined the strength values that would be expected for each condition at high clustering values. At the lower PB_{RSA}-rest bound we observe weaker network connections between highly clustered nodes during abstinence compared to normal drinking, while at the upper PB_{RSA}-rest bound we observe stronger connections between highly clustered nodes following abstinence.

Although it did not reach statistical significance, a notable corresponding trend was evident in the relationship between drinking state, PB_{RSA}-rest, and global efficiency ($\beta = -0.00518$, $p = 0.1087$, Supp. Fig. 2). The difference in the relationship between connection strength and global efficiency change in opposite directions across drinking state between the upper and lower bounds of PB_{RSA}-rest. At the lower bound the difference in slope increased as global efficiency increased, with greater strength at more globally efficient nodes observed in abstinence compared to normal drinking. At the upper bound the difference in slope decreased as global efficiency increased, and greater strength in highly globally efficient nodes was again observed n abstinence compared to normal drinking.

Suppl. Table 1 provides the key results for the connection strength mixed-model. There was a significant main effect of drinking state on connection strength, such that

greater average connection strength was found during normal dinking compared to abstinence (β = 1.2340, $p < 0.0001$). The significant main effects of PB_{RSA}-rest indicated that, on average, participants with higher PB_{RSA}-rest had lower network connection strength (β = -0.00994, $p = 0.0220$). There was an interaction between drinking state and PB_{RSA}-rest that did not reach statistical significance, but exhibited a notable positive trend (β = 0.01180, $p = 0.0553$). Clustering (β = 0.06825, $p < 0.0001$) and global efficiency (β = 0.02923, $p < 0.0001$) were both statistically significant positive predictors of connection strength. Interactions between drinking state and these network topological features were not statistically significant. There was an interaction of PB_{RSA}-rest with clustering (β = -0.00532, $p = 0.0004$), but not with global efficiency.

2.2 Full Findings

Full results for the connection strength model can be found in Supp. Table 2. All four network features of interest associated significantly with connection strength. Clustering (β = 0.06825, $p < 0.0001$) and global efficiency (β = 0.02923, $p < 0.0001$) were positively associated with connection strength, while degree (β = -0.04430, $p < 0.0001$) and modularity (β = -0.01518, $p < 0.0001$) were negatively associated with connection strength. The relationship between PB_{RSA}-react scores and connection strength approached significance (β = 0.007051, $p < 0.0898$), in the inverse direction of the relationship between connection strength and PB_{RSA}-rest.

Significant interactions between connection strength and PB_{RSA}-rest and react were seen with clustering (β = -0.05132, $p = 0.0406$) and global efficiency (β = -0.05132, $p = 0.0440$), although the relationship between PB_{RSA}-rest and react was not significant. When examining the clustering relationship (Supp. Fig. 3), participants at the lower bound of PB_{RSA}-rest but the upper-bound of PB_{RSA}-react showed the strongest relationship between clustering and connection strength. Participants at the lower bound of PB_{RSA}-react, both with high and low PB_{RSA}-rest, showed similar relationships between clustering and connection strength. The weakest connections between highly clustered nodes was observed in participants at the upper bound of PB_{RSA}-rest and react. For the global efficiency relationship (Supp. Fig. 4), participants at the lower bound of both PB_{RSA}-rest and react, as well as those at the upper-bound of both PB_{RSA}-rest and react showed the strongest relationship between global efficiency and connections strength. The weakest connections between highly globally efficient nodes were observed in at the upper PB_{RSA}-rest bound, but the lower PB_{RSA}-react bound.

Full results for the probability model can be found in Supp. Table 3. Clustering (β = 0.3214, $p < 0.0001$) and degree (β = 0.1480, $p < 0.0001$) showed significant positive associations with connection probability, while global efficiency showed a significant negative relationship with connection probability (β = -0.3050, $p < 0.0001$). Spatial Distance (β = 0.3597, $p < 0.0001$) and Spatial Distance^2 (β = -0.1346, $p < 0.0001$) were significantly associated with connection probability. Drinking state was positively associated with probability (β = 0.1803, $p < 0.0001$), indicating greater average probability of connection was found during normal drinking compared to abstinence. Finally, a significant interaction was found between degree and PB_{RSA}-rest with connection probability (β = 0.04649, $p = 0.0389$), such that the higher degree observed with greater probability of connections was even greater at higher PB_{RSA}-rest values.

3. Supplementary Tables and Figures

3.1 Supplementary Tables

Supplementary Table S1. Relevant mixed-model strength results. Significant effects and interactions are bolded.

Effect	Estimate	Standard Error	p-value
**Drinking State**	**0.2340**	**0.004659**	**<0.0001**
**PB_{RSA}-rest**	**-0.00994**	**0.004340**	**0.0220**
Drinking State*PB_{RSA}-rest	0.01180	0.006157	0.0553
**Clustering Coefficient**	**0.06825**	**0.002450**	**<0.0001**
**Global Efficiency**	**0.02923**	**0.002528**	**<0.0001**
Drinking State*Clustering Coefficient	0.001887	0.003163	0.5509
Drinking State*Global Efficiency	-0.00456	0.003046	0.1344
**PB_{RSA}-rest*Clustering Coefficient**	**-0.00532**	**0.002651**	**0.0449**
PB_{RSA}-rest*Global Efficiency	0.000041	0.002713	0.9879
**Drinking State*PB_{RSA}-rest*Clustering Coefficient**	**0.01186**	**0.003357**	**0.0004**
Drinking State*PB_{RSA}-rest*Global Efficiency	-0.00518	0.003357	0.1087
**Spatial Distance**	**-0.05132**	**0.001100**	**<0.0001**
**Spatial Distance^2**	**0.02890**	**0.000537**	**<0.0001**
Age	-0.00116	0.003233	0.7187
Sex	-0.00012	0.005968	0.9843
BMI	-0.00348	0.003519	0.3221

Supplementary Table S2. Full mixed-effects model results – strength.

Effect	Estimate	Standard Error	Adaptive FDR p-values
Intercept	0.2340	0.004659	< 0.0001
Drinking State	-0.01502	0.005259	0.0133
PB_{RSA}-rest	-0.00994	0.004340	0.0616
PB_{RSA}-react	0.007051	0.004156	0.1676
Clustering Coefficient	0.06825	0.002450	< 0.0001
Global Efficiency	0.02923	0.002528	< 0.0001
Degree	-0.04430	0.001256	< 0.0001
Modularity	-0.01518	0.003053	< 0.0001
Age	-0.00116	0.003233	0.7187
Sex	-0.00012	0.005968	0.9843
BMI	-0.00348	0.003519	0.3786
Distance	-0.05132	0.001100	< 0.0001
Distance^2	0.02890	0.000537	< 0.0001
Clustering Coefficient*PB_{RSA}-rest*PB_{RSA}-react	-0.00223	0.001092	0.0967
Global Efficiency*PB_{RSA}-rest*PB_{RSA}-react	0.001999	0.000993	0.0967
Degree*PB_{RSA}-rest*PB_{RSA}-react	-0.00051	0.000561	0.3874
Modularity*PB_{RSA}-rest*PB_{RSA}-react	-0.00570	0.004092	0.2291
Clustering Coefficient*Drinking State*PB_{RSA}-rest	0.01186	0.003357	0.0014
Global Efficiency*Drinking State*PB_{RSA}-rest	-0.00518	0.003226	0.1749
Degree*Drinking State*PB_{RSA}-rest	-0.00120	0.001734	0.4897
Modularity*Drinking State*PB_{RSA}-rest	-0.00138	0.007352	0.8511

Clustering Coefficient*Drinking State*PB$_{RSA}$-react	-0.00329	0.003343	0.3786
Global Efficiency*Drinking State*PB$_{RSA}$-react	0.002779	0.003215	0.3874
Degree*Drinking State*PB$_{RSA}$-react	0.001632	0.001730	0.3871
Modularity*Drinking State*PB$_{RSA}$-react	-0.01339	0.008060	0.1692
Clustering Coefficient*PB$_{RSA}$-rest	-0.00532	0.002651	0.0967
Global Efficiency*PB$_{RSA}$-rest	0.000041	0.002713	0.9879
Degree*PB$_{RSA}$-rest	-0.00049	0.001351	0.7183
Modularity*PB$_{RSA}$-rest	0.003155	0.003607	0.3874
Clustering Coefficient*PB$_{RSA}$-react	0.000271	0.002731	0.9209
Global Efficiency*PB$_{RSA}$-react	0.001526	0.002783	0.5835
Degree*PB$_{RSA}$-react	-0.00154	0.001399	0.3530
Modularity*PB$_{RSA}$-react	0.006400	0.005891	0.3530
Clustering Coefficient*Drinking State	0.001887	0.003163	0.5509
Global Efficiency*Drinking State	-0.00456	0.003046	0.1981
Degree*Drinking State	0.000871	0.001643	0.5961
Modularity*Drinking State	-0.00044	0.005495	0.9367
Drinking State*PB$_{RSA}$-rest	0.01180	0.006157	0.1106
Drinking State*PB$_{RSA}$-react	-0.00886	0.005583	0.1749
PB$_{RSA}$-rest* PB$_{RSA}$-react	-0.00021	0.002646	0.9366

Supplementary Table S3. Full mixed-effects model results – probability.

**Effect**	**Estimate**	**Standard Error**	**Adaptive FDR p-values**
Intercept	0.1803	0.03466	< 0.0001
Drinking State	-0.00310	0.04010	0.9383
PB$_{RSA}$-rest	0.01808	0.03196	0.7998
PB$_{RSA}$-react	0.01719	0.03062	0.7998
Clustering Coefficient	0.3214	0.03946	< 0.0001
Global Efficiency	-0.3051	0.03295	< 0.0001
Degree	0.1480	0.02142	< 0.0001
Modularity	-0.02214	0.02248	0.7304
Age	-0.00490	0.02444	0.8502
Sex	0.05785	0.04534	0.6965
BMI	-0.02645	0.02663	0.7304
Distance	0.3597	0.01688	< 0.0001
Distance^2	-0.1346	0.007011	< 0.0001
Clustering Coefficient*PB$_{RSA}$-rest*PB$_{RSA}$-react	-0.01727	0.01506	0.6965
Global Efficiency*PB$_{RSA}$-rest*PB$_{RSA}$-react	0.002236	0.01408	0.8738
Degree*PB$_{RSA}$-rest*PB$_{RSA}$-react	0.007827	0.01016	0.7998
Modularity*PB$_{RSA}$-rest*PB$_{RSA}$-react	0.02910	0.03130	0.7466
Clustering Coefficient*Drinking State*PB$_{RSA}$-rest	0.06126	0.04975	0.6965
Global Efficiency*Drinking State*PB$_{RSA}$-rest	-0.02835	0.04411	0.7998
Degree*Drinking State*PB$_{RSA}$-rest	-0.03307	0.03111	0.7304

Modularity*Drinking State*PB_{RSA}-rest	-0.01012	0.05689	0.8588
Clustering Coefficient*Drinking State*PB_{RSA}-react	0.01006	0.04971	0.8502
Global Efficiency*Drinking State*PB_{RSA}-react	-0.00360	0.04409	0.9349
Degree*Drinking State*PB_{RSA}-react	-0.01732	0.03110	0.7998
Modularity*Drinking State*PB_{RSA}-react	-0.02415	0.06135	0.8502
Clustering Coefficient*PB_{RSA}-rest	0.002919	0.04219	0.9448
Global Efficiency*PB_{RSA}-rest	-0.01796	0.03530	0.8145
Degree*PB_{RSA}-rest	0.04652	0.02302	0.2224
Modularity*PB_{RSA}-rest	-0.03170	0.02644	0.6965
Clustering Coefficient*PB_{RSA}-react	-0.02654	0.04336	0.7998
Global Efficiency*PB_{RSA}-react	0.02398	0.03655	0.7998
Degree*PB_{RSA}-react	0.005727	0.02403	0.8502
Modularity*PB_{RSA}-react	0.008263	0.04331	0.8502
Clustering Coefficient*Drinking State	-0.05454	0.04713	0.6965
Global Efficiency*Drinking State	0.04795	0.04181	0.6965
Degree*Drinking State	0.01711	0.02950	0.7998
Modularity*Drinking State	-0.01085	0.04249	0.8502
Drinking State*PB_{RSA}-rest	-0.03603	0.04698	0.7998
Drinking State*PB_{RSA}-react	-0.01413	0.04272	0.8502
PB_{RSA}-rest* PB_{RSA}-react	0.007968	0.01971	0.8502

Supplementary Table S4. Differences in average survey responses preceding MRI scanning across drinking states.

	Normal Drinking	Abstinence	t-value	p-value
**Alcohol Craving Experience (ACE) Questionnaire**	14.1 (13.5)	14.5 (10.9)	-0.247	0.806
**Perceived Stress Scale (PSS)**	30.8 (2.8)	30.3 (2.3)	1.404	0.168
**State Trait Anxiety Inventory (STAI)**	45.9 (3.9)	45.9 (3.4)	0.092	0.927
**Mindful Attention Awareness Scale (MAAS)**	7.8 (5.2)	7.6 (5.6)	0.272	0.787
**Freiburg Mindfulness Scale**	40.7 (5.6)	41.5 (5.9)	-1.548	0.130

3.2 Supplementary Figures

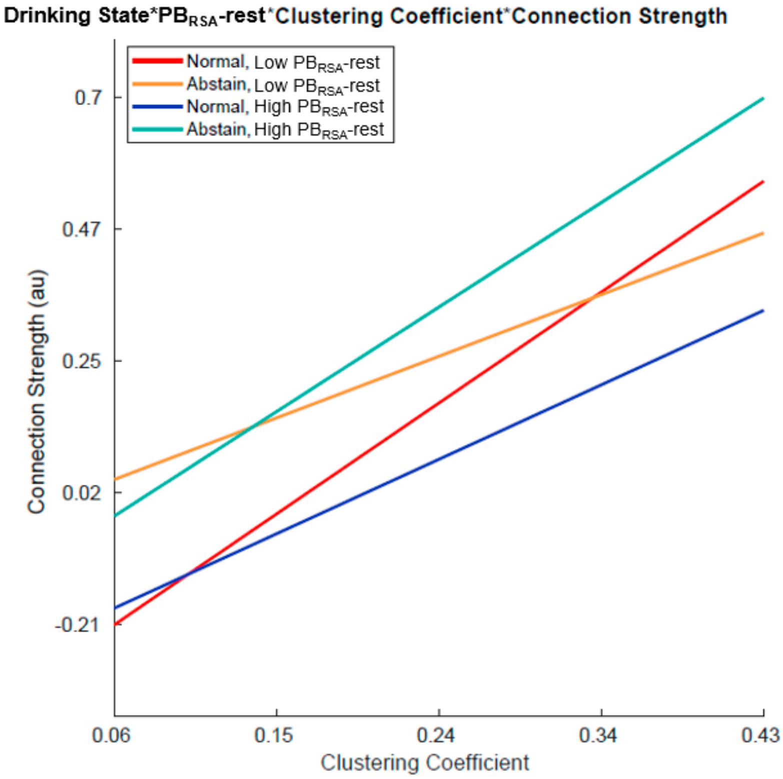

Figure S1. Graphical representation of the relationship between connection strength and clustering coefficient across drinking states, divided by the upper and lower bounds of PB_{RSA}-rest values, meant only for illustrative purposes. Because PB_{RSA} is a continuous variable, the 3-way interactions form 3-dimentional planes and cannot easily be graphed. For visualization purposes, the upper and lower bounds of PB_{RSA}-rest values were used in the regression equations in conjunction with the dichotomous drinking state variable to create this graph.

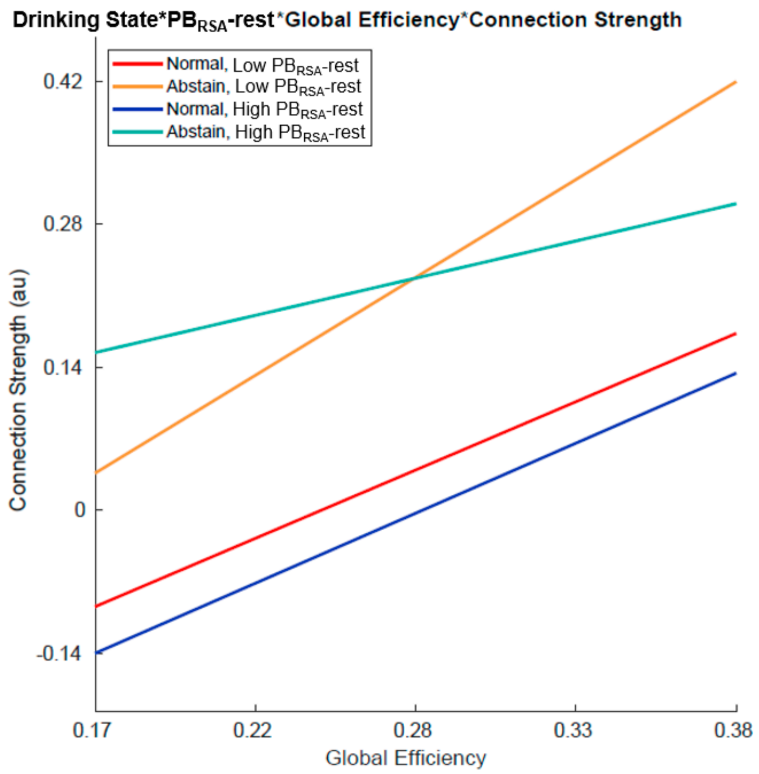

Figure S2. Graphical representation of the relationship between connection strength and global efficiency across drinking states, divided by PB$_{RSA}$-rest values (approaching significance), meant only for illustrative purposes. Because PB$_{RSA}$ is a continuous variable, the 3-way interactions form 3-dimentional planes and cannot easily be graphed. For visualization purposes, the upper and lower bounds of PB$_{RSA}$-rest values were used in the regression equations in conjunction with the dichotomous drinking state variable to create this graph.

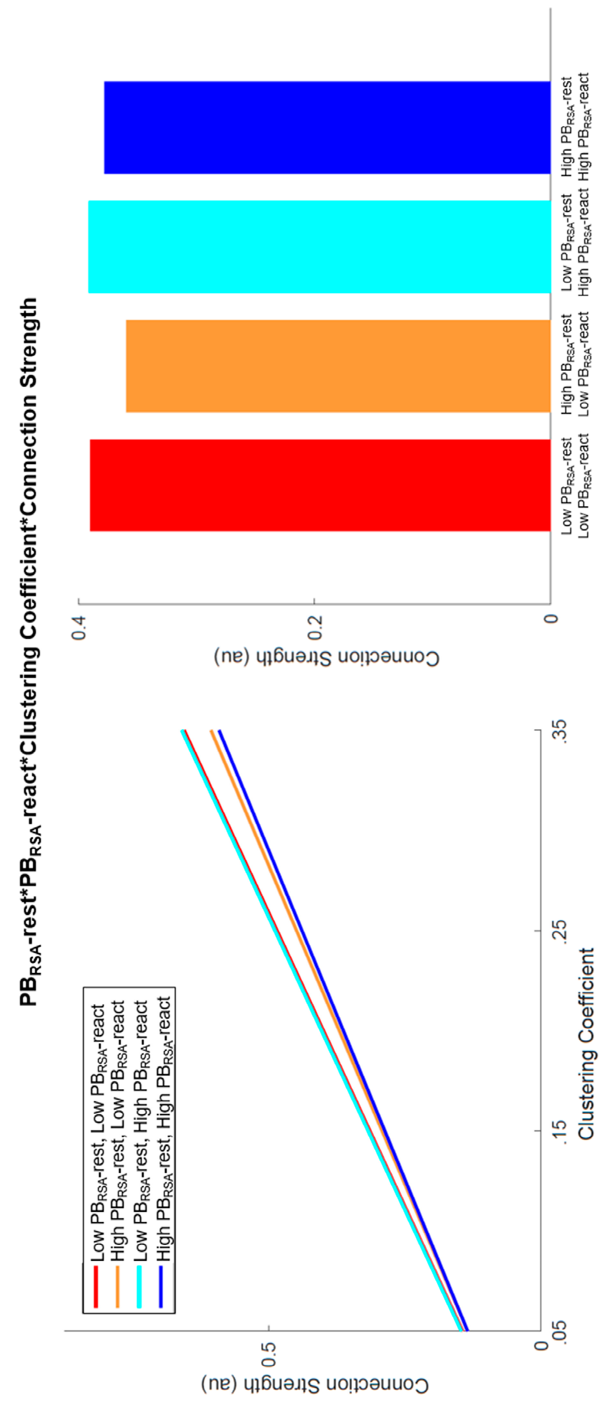

Figure S3. Graphical representation of the relationship between connection strength and clustering coefficient when accounting for both resting and reactive PBRSA, meant only for illustrative purposes. Because PBRSA is a continuous variable, the 3-way interactions form 3-dimentional planes and cannot easily be graphed. For visualization purposes, the upper and lower bounds of PBRSA-rest and PBRSA-react values were used in the regression equations to create this graph. A depiction of the regression equation (as included in Supplementary Figures 1 and 2) as well as the slopes of the four conditions (as seen in the main body of the manuscript) are included.

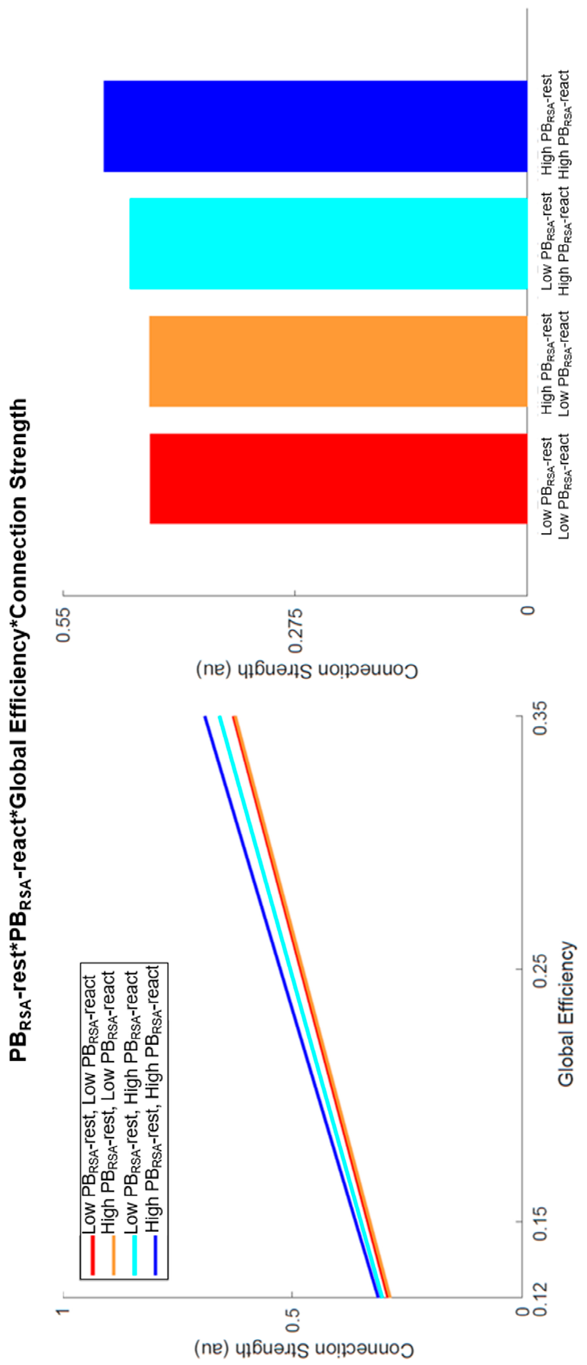

Figure S4. Graphical representation of the relationship between connection strength and global efficiency when accounting for both resting and reactive PBRSA, meant only for illustrative purposes. Because PBRSA is a continuous variable, the 3-way interactions form 3-dimentional planes and cannot easily be graphed. For visualization purposes, the upper and lower bounds of PBRSA-rest and PBRSA-react values were used in the regression equations to create this graph. A depiction of the regression equation (as included in Supplementary Figures 1 and 2) as well as the slopes of the four conditions (as seen in the main body of the manuscript) are included.

References

[1] 56. Simpson, S.L. and P.J. Laurienti, *A two-part mixed-effects modeling framework for analyzing whole-brain network data.* Neuroimage, 2015. **113**: p. 310-319.

[2] 57. Simpson, S.L., M. Bahrami, and P.J. Laurienti, *A mixed-modeling framework for analyzing multitask whole-brain network data.* Network Neuroscience, 2019. **3**(2): p. 307-324.

[3] 59. Benjamini, Y. and Y. Hochberg, *On the adaptive control fo the false discovery rate in multiple testing with independent statistics.* J Educ Behav Stat, 2000. **25**(1): p. 60-83.

[4] 55. Bahrami, M., P.J. Laurienti, and S.L. Simpson, *A MATLAB toolbox for multivariate analysis of brain networks.* Human Brain Mapping, 2018. **40**(1): p. 175-186.

[5] 81. Rubinov, M. and O. Sporns, *Complex network measures of brain connectivity: uses and interpretations.* Neuroimage, 2010. **52**(3): p. 1059-69.

[6] 60. Newman, M.E. and M. Girvan, *Finding and evaluating community structure in networks.* Phys Rev E Stat Nonlin Soft Matter Phys, 2004. **69**(2): p. 026113.

[7] 61. Demeersman, R.E., *Aging as a moderator of respiratory sinus arrhythmia.* Journals of Gerontology, 1993. **48**(2): p. B74-78.

[8] 62. Seo, D., et al., *Sex differences in neural responses to stress and alcohol context cues.* Human Brain Mapping, 2011. **32**(11): p. 1998-2013.

[9] 39. Wang, M.Q., et al., *Acute alcohol intoxication, body composition, and pharmacokinetics.* Pharmacology Biochemistry and Behavior, 1992. **43**(2): p. 641.

[10] 82. Friedman, E.J., et al., *Stochastic geometric network models for groups of functional and structural outcomes.* Neuroimage, 2014. **101**: p. 473-484.
